# Natural course and risk factors of moyamoya disease with unruptured intracranial aneurysm

**DOI:** 10.3389/fneur.2023.1115909

**Published:** 2023-02-09

**Authors:** Ri-Miao Yang, Fang-Bin Hao, Bo Zhao, Qian Zhang, Dan Yu, Zheng-Xing Zou, Gan Gao, Qing-Bao Guo, Xu-Xuan Shen, He-Guan Fu, Si-Meng Liu, Min-Jie Wang, Jing-Jie Li, Cong Han

**Affiliations:** ^1^Department of Neurosurgery, Chinese PLA General Hospital, Beijing, China; ^2^Chinese PLA Medical School, Beijing, China; ^3^307 Clinical College of Anhui Medical University, Beijing, China

**Keywords:** moyamoya disease, intracranial aneurysms, natural course, risk factors, follow-up

## Abstract

**Background and objective:**

The natural course and risk factors of moyamoya disease (MMD) associated with unruptured intracranial aneurysms involving stenosed parental arteries are scarcely studied. This study aimed to elucidate the natural course of MMD and its associated risk factors in patients with MMD with unruptured aneurysms.

**Methods:**

Between September 2006 and October 2021, patients with MMD with intracranial aneurysms at our center were examined. The natural course, clinical features, radiological features, and follow-up outcomes after revascularization were analyzed.

**Results:**

This study included 42 patients with MMD with intracranial aneurysms (42 aneurysms). The age distribution of MMD cases ranged from 6 to 69 years, with four children (9.5%) and 38 adults (90.5%). A total of 17 male and 25 female subjects were included (male-to-female ratio: 1:1.47). The first symptom was cerebral ischemia in 28 cases, and cerebral hemorrhage occurred in 14 cases. There were 35 trunk aneurysms and seven peripheral aneurysms. There were 34 small aneurysms (<5 mm) and eight medium aneurysms (5–15 mm). During the average clinical follow-up period of 37.90 ± 32.53 months, there was no rupture or bleeding from aneurysms. Twenty-seven of these patients underwent a cerebral angiography review, in which it was found that one aneurysm had enlarged, 16 had remained unchanged, and 10 had shrunk or disappeared. A correlation exists between the reduction or disappearance of aneurysms and the progression of the Suzuki stages of MMD (*P* = 0.015). Nineteen patients underwent EDAS on the aneurysm side, and nine aneurysms disappeared, while eight patients did not undergo EDAS on the aneurysm side and one aneurysm disappeared.

**Conclusion:**

The risk of rupture and hemorrhage of unruptured intracranial aneurysms is low when the parent artery already has stenotic lesions, thus, direct intervention may not be necessary for such aneurysms. The progression of the Suzuki stage of moyamoya disease may play a role in the shrinkage or disappearance of the aneurysms, thereby decreasing the risk of rupture and hemorrhage. Encephaloduroarteriosynangiosis (EDAS) surgery may also help promote atrophy or even the disappearance of the aneurysm, thus reducing the risk of further rupture and bleeding.

## 1. Introduction

Moyamoya disease (MMD), commonly known as the abnormal vascular network disease of the skull base, is characterized by the gradual stenosis of bilateral internal carotid artery terminals and/or the stems of the middle cerebral artery and anterior cerebral artery, progressive narrowing of the arterial lumen, and the consequent compensatory expansion of perforating arteries at the base of the skull (1). Thus, intracranial aneurysms are a common occurrence among patients with moyamoya disease. The incidence of intracranial aneurysms in the general population ranges from 1 to 3%, while the probability of aneurysms is 3.4%−15% among patients with MMD (2, 3). This increased risk may be related to the hemodynamic and vascular pathological changes seen in MMD. MMD combined with an aneurysm is one of the important causes of intracranial hemorrhage (4).

Moyamoya disease combined with an unruptured intracranial aneurysm presents surgeons with an exceptional challenge in the management of aneurysms (5–8). As to aneurysms that have already ruptured, aggressive interventions are necessary. As open surgery carries the risk of destroying compensatory vascular collaterals, resulting in complications, endovascular treatment (EVT) has become the mainstream treatment modality for MMD-associated aneurysms. However, its utility in unruptured aneurysms remains controversial. Previous studies have reported that aneurysms, supplied by a parent artery that is embedded in an MMD lesion, could atrophy and disappear with the progressive narrowing of the parent artery (9); however, little information is available on the clinical characteristics and long-term prognosis of patients with these concurrent conditions. Accordingly, this study aimed to survey and observe the natural course of intracranial aneurysms in patients with MMD, analyze the factors that may affect changes in these aneurysms, and assess their risk of rupture and bleeding.

## 2. Materials and methods

### 2.1. Patient data

This study was approved by the Fifth Medical Center of the PLA General Hospital Research Ethics Committee (no. KY-2022-9-69-1). The clinical data of 42 patients with MMD and untreated intracranial aneurysms were collected at our center from September 2006 to October 2021. Clinical features such as the age of onset, gender, modified Rankin Scale (mRS), hypertension, diabetes mellitus, hyperlipemia, hyperhomocysteinemia, and anemia were collected.

**Figure 1:**
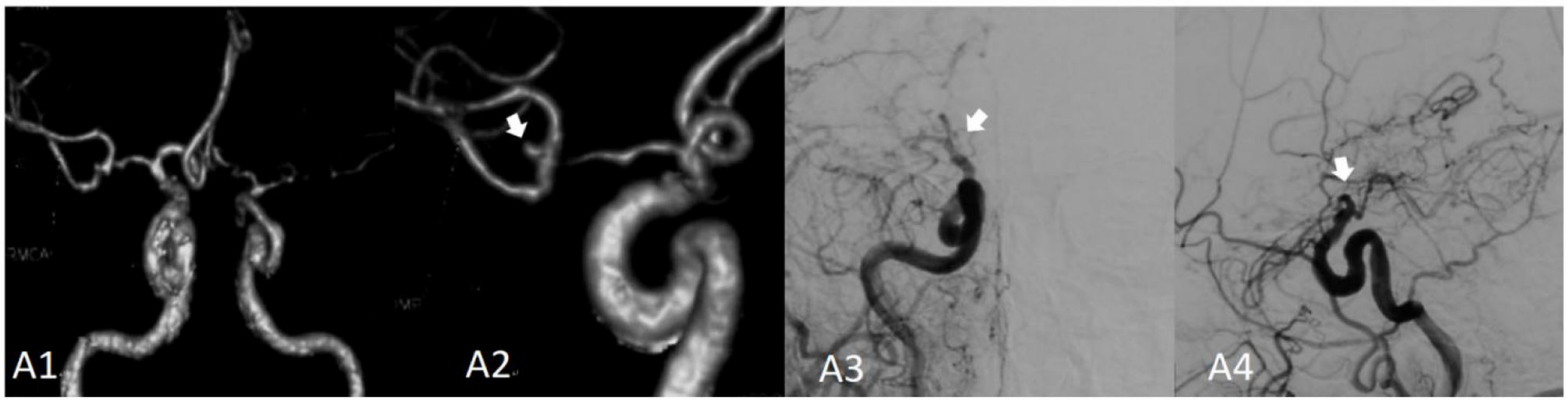
Moyamoya disease combined with a bifurcation aneurysm of the right middle cerebral artery. **(A1)** Shows the computed tomography angiography (CTA) image of the bilateral internal carotid artery in 2014; **(A2)** shows the locally enlarged CTA of the right internal carotid artery; the aneurysm is indicated by the white arrow. **(A3)** Presents the right internal carotid artery angiography (red arrow) image. **(A4)** Presents the right internal carotid artery angiography image; the distal end of the internal carotid artery is not occluded, and the parent artery and aneurysm have disappeared.

Patients were selected based on the following inclusion criteria: (1) the presence of MMD diagnosed *via* the 2021 diagnostic criteria of MMD (10) in Japan; (2) the presence of MMD combined with an intracranial aneurysm; (3) intracranial aneurysm that has not ruptured; and (4) it is found that the parent artery of the aneurysm is narrowed (Figure 1).

The following patients were excluded: (1) patients with MMD and intracranial aneurysm after having completed aneurysm surgery and (2) patients with other cerebrovascular lesions (such as vascular malformations and arteriovenous fistulas).

### 2.2. Intracranial aneurysms and subclassifications

Diagnoses of intracranial aneurysms were made using conventional cerebral digital subtraction angiography (DSA). DSA images were reviewed and interpreted independently by at least two neurologists, and the results were confirmed by another neurosurgeon at our institution. The results of the angiograms, including aneurysm size, morphology (saccular, fusiform, or dissecting), and location were recorded. Patients with aneurysms were divided into two groups: (1) trunk aneurysms and (2) peripheral aneurysms (11). Aneurysm sizes, recorded as the maximum two-dimensional angiographic dimensions, were assigned to four categories: (1) 5 mm, (2) 5–15 mm, (3) 15–25 mm, or (4) >25 mm (4).

### 2.3. Surgical treatment

Thirty-one patients receive 56 encephaloduroarteriosynangiosis (EDAS) procedures performed by the same neurosurgeon in our department. Twenty-two patients underwent bilateral EDAS of the superficial temporal artery.

### 2.4. Clinical follow-up

Clinical follow-up was conducted through outpatient, inpatient follow-up, and telephone calls to know whether the patient had a hemorrhagic stroke (12). Aneurysm changes were observed according to the DSA follow-up, including enlargement, stability, shrinkage, or disappearance. Neurological outcome was evaluated using the modified Rankin scale (mRS).

### 2.5. Statistical analysis

All statistical analyses were performed with the statistical software packages R (www.R-project.org, The R Foundation) and Free Statistics software version 1.7 (13). Qualitative factors were analyzed using a standard chi-square test or Fisher's exact test, and quantitative factors were analyzed using an unpaired Student's *t*-test or Mann–Whitney *U*-test. Variables associated with aneurysm formation included age of onset, sex, hypertension, diabetes mellitus, hyperlipemia, hyperhomocysteinemia, and anemia. Variables associated with aneurysm risk included aneurysm size, morphology, and location. All values were expressed as the mean standard deviation. Statistical significance was set at *P* < 0.05.

## 3. Results

### 3.1. Demographics and clinical presentation

A total of 42 cases in this study accounted for 41.6% (42/101) of patients with MMD and complicated aneurysms during the study period. A total of 17 men and 25 women were included (male-to-female ratio: 1:1.47). The patients' ages ranged from 6 to 69 years, including 38 adults and four children. There were 14 cases of cerebral hemorrhage (33.3%), including eight cases of intraventricular hemorrhage, one case of subarachnoid hemorrhage, and five cases of cerebral parenchymal hemorrhage. There were 28 cases of cerebral ischemia (66.6%), including two cases of cerebral infarction, 16 cases of transient ischemic attack, two cases of headaches, seven cases of vertigo, and one case of epilepsy. A total of 35 trunk aneurysms occurred in this group, which was comprised of 20 internal carotid aneurysms, one anterior cerebral artery, three anterior communicating artery aneurysms, two middle cerebral artery aneurysms, four posterior cerebral artery aneurysms, and five basilar artery aneurysms. There were also seven peripheral aneurysms, including five anterior choroidal aneurysms, one posterior choroidal aneurysm, and one in the moyamoya arteries. In this group, small aneurysms accounted for 34 cases and medium aneurysms accounted for eight cases. Compared with trunk aneurysms, peripheral aneurysms had a higher percentage of cerebral hemorrhage as an initial MMD symptom and a lower percentage of hypertension (Table 1).

**Table 1:** Clinical features of 42 patients with MMD with intracranial aneurysms.

**Variables**	**Total (*n* = 42)**	**Peripheral aneurysm (*n* = 7)**	**Trunk aneurysm (*n* = 35)**	***P*-value**	**Statistic test**
Age (years)	38.6 ± 14.1	39.4 ± 7.4	38.4 ± 15.2	0.866	0.029
Female, sex, *n* (%)	25 (59.5)	7 (100)	18 (51.4)	0.03	Fisher
Initial clinical manifestations, *n* (%)				0.519	Fisher
TIA	12 (28.6)	2 (28.6)	10 (28.6)	
Infarction	6 (14.3)	0 (0)	6 (17.1)		
Hemorrhage	14 (33.3)	4 (57.1)	10 (28.6)		
Others	10 (23.8)	1 (14.3)	9 (25.7)		
Hypertension, *n* (%)	13 (31.0)	1 (14.3)	12 (34.3)	0.405	Fisher
Diabetes, *n* (%)	2 (4.8)	0 (0)	2 (5.7)	1	Fisher
Hyperlipemia, *n* (%)	4 (9.5)	0 (0)	4 (11.4)	1	Fisher
Hyperhomocysteinemia, *n* (%)	1 (2.4)	0 (0)	1 (2.9)	1	Fisher
Anemia, *n* (%)	5 (11.9)	3 (42.9)	2 (5.7)	0.026	Fisher
mRS at admission, *n* (%)				1	Fisher
1	28 (66.7)	5 (71.4)	23 (65.7)	
2	12 (28.6)	2 (28.6)	10 (28.6)		
3	1 (2.4)	0 (0)	1 (2.9)		
4	1 (2.4)	0 (0)	1 (2.9)		
Suzuki stage on aneurysm side, *n* (%)				0.683	Fisher
0	3 (7.1)	0 (0)	3 (8.6)	
1	5 (11.9)	0 (0)	5 (14.3)		
2	4 (9.5)	0 (0)	4 (11.4)		
3	10 (23.8)	3 (42.9)	7 (20)		
4	13 (31.0)	2 (28.6)	11 (31.4)		
5	4 (9.5)	1 (14.3)	3 (8.6)		
6	3 (7.1)	1 (14.3)	2 (5.7)		
Size of aneurysm, *n* (%)				0.601	Fisher
Small (<5 mm)	34 (81.0)	5 (71.4)	29 (82.9)		
Medium (5–15 mm)	8 (19.0)	2 (28.6)	6 (17.1)		

MMD, moyamoya disease; TIA, transient ischemic attacks; mRS, modified Rankin Scale.

### 3.2. Follow-up

Thirty of the 42 patients were followed up. Among the 30 patients monitored during the average follow-up period of 37.90 ± 32.53 months, the mRS scores of 23 patients decreased, the mRS scores of five patients remained unchanged, and the mRS scores of two patients increased. Both patients with increased mRS scores suffered from basal ganglia cerebral hemorrhage and non-aneurysm rupture hemorrhage. Of the 22 aneurysms localized in the anterior circulation, eight were located distal to the posterior communicating artery and five aneurysms decreased or disappeared (Table 2).

**Table 2:** Characteristics of 27 MMD patients with intracranial aneurysms on follow-up.

**Variables**	**Total (*n* = 27)**	**Aneurysm shrunk or disappeared (*n* = 10)**	**Aneurysm unchanged (*n* = 17)**	***P***	**Statistical test**
Age (years)	37.6 ± 12.4	39.3 ± 10.9	36.5 ± 13.4	0.585	0.306
Female, sex, n (%)	16 (59.3)	8 (80)	8 (47.1)	0.124	Fisher
Initial clinical manifestations, n (%)				0.613	Fisher
TIA	9 (33.3)	5 (50)	4 (23.5)		
Infarction	4 (14.8)	1 (10)	3 (17.6)		
Haemorrhage	8 (29.6)	2 (20)	6 (35.3)		
Others	6 (22.2)	2 (20)	4 (23.5)		
Hypertension, n (%)	9 (33.3)	4 (40)	5 (29.4)	0.683	Fisher
Diabetes, n (%)	1 (3.7)	0 (0)	1 (5.9)	1	Fisher
Hyperlipemia, n (%)	3 (11.1)	1 (10)	2 (11.8)	1	Fisher
Hyperhomocysteinemia, n (%)	1 (3.7)	0 (0)	1 (5.9)	1	Fisher
Size of aneurysm, n (%)				0.128	Fisher
Small (<5 mm)	23 (85.2)	7 (70)	16 (94.1)		
Medium (5–15 mm)	4 (14.8)	3 (30)	1 (5.9)		
EDAS on the aneurysm side, n (%)	19 (70.4)	9 (90)	10 (58.8)	0.190	Fisher
Location of aneurysm, n (%)				0.078	Fisher
The distal end of the anterior circulation	8 (29.6)	5 (50)	3 (17.6)		
The proximal end of the anterior circulation	13 (48.1)	2 (20)	11 (64.7)		
PCA	6 (22.2)	3 (30)	3 (17.6)		
Trunk aneurysm, n (%)	21 (77.8)	7 (70)	14 (82.4)	0.638	Fisher
Progression of Suzuki grade, n (%)	6 (22.2)	5 (50)	1 (5.9)	0.015	Fisher

MMD, moyamoya disease; TIA, transient ischemic attacks; PCA, vertebrobasilar artery or posterior cerebral artery.

**Figure 2:**
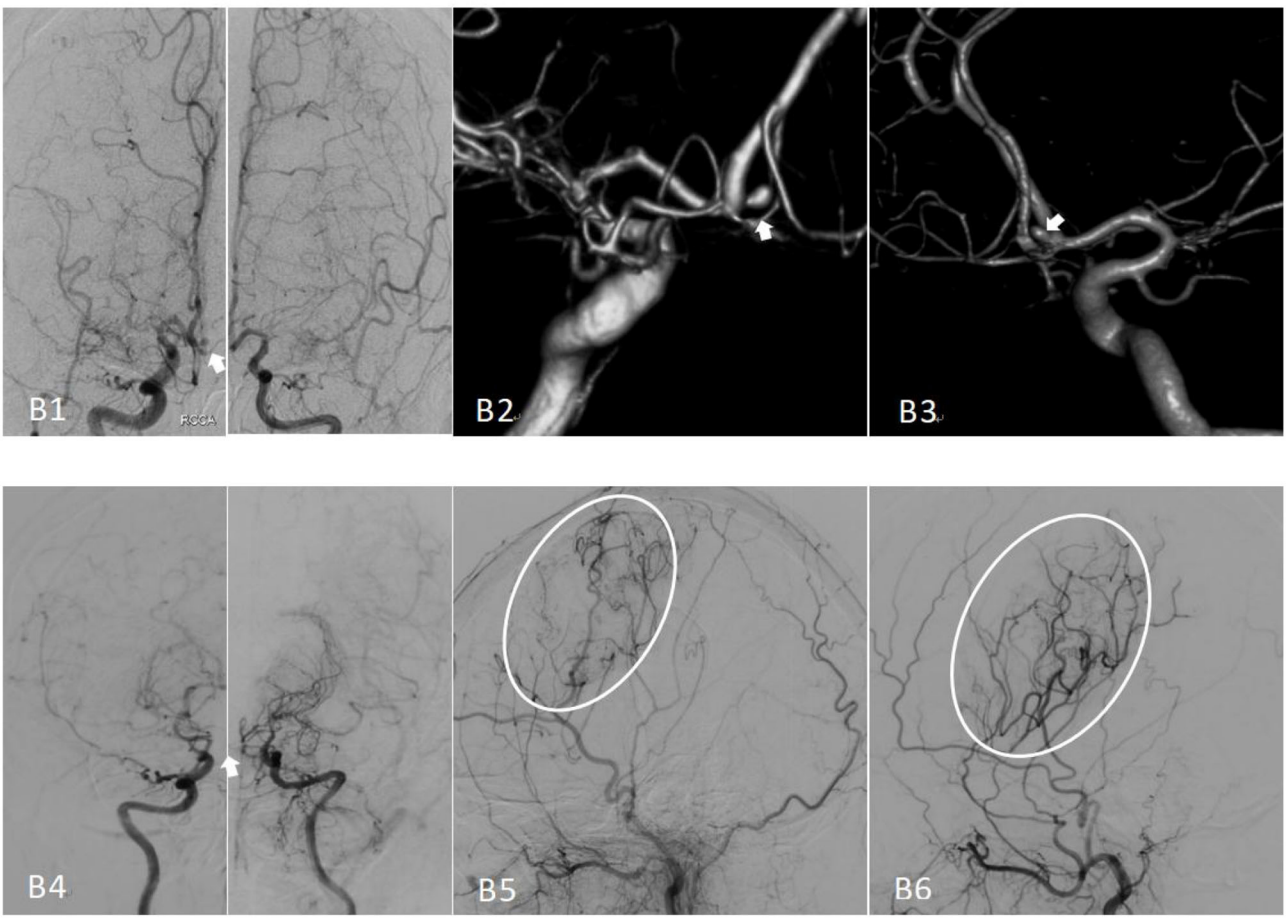
Preoperative and postoperative cerebral angiography of moyamoya disease treated with encephaloduroarteriosynangiosis (EDAS). **(B1)** The bilateral preoperative internal carotid angiography (positive) image, with an anterior communicating artery aneurysm indicated by the white arrow. **(B2, B3)** Images of the preoperative angiography. **(B4)** The bilateral internal carotid angiography image, Suzuki stage II–V; note that the aneurysm has disappeared. **(B5)** An image of the right external carotid angiography (lateral) and vascular reconstruction. **(B6)** The postoperative left external carotid angiography (lateral) image.

In this study, 27 cases were re-examined by angiography, and the mean follow-up time for repeat angiography was 20.50 ± 18.28 months. Among the 27 cases of aneurysms, one case of aneurysm enlarged, 16 cases of aneurysms remained unchanged, and 10 cases of aneurysms became smaller or disappeared. Among the 10 cases with downgraded aneurysms, six of them were trunk aneurysms, four were peripheral aneurysms, eight were located in the anterior circulatory system, and two were located in the posterior circulatory system. Among the follow-up patients, five aneurysms disappeared in the six patients with the developed Suzuki stage (Figure 2).

**Figure 3:**
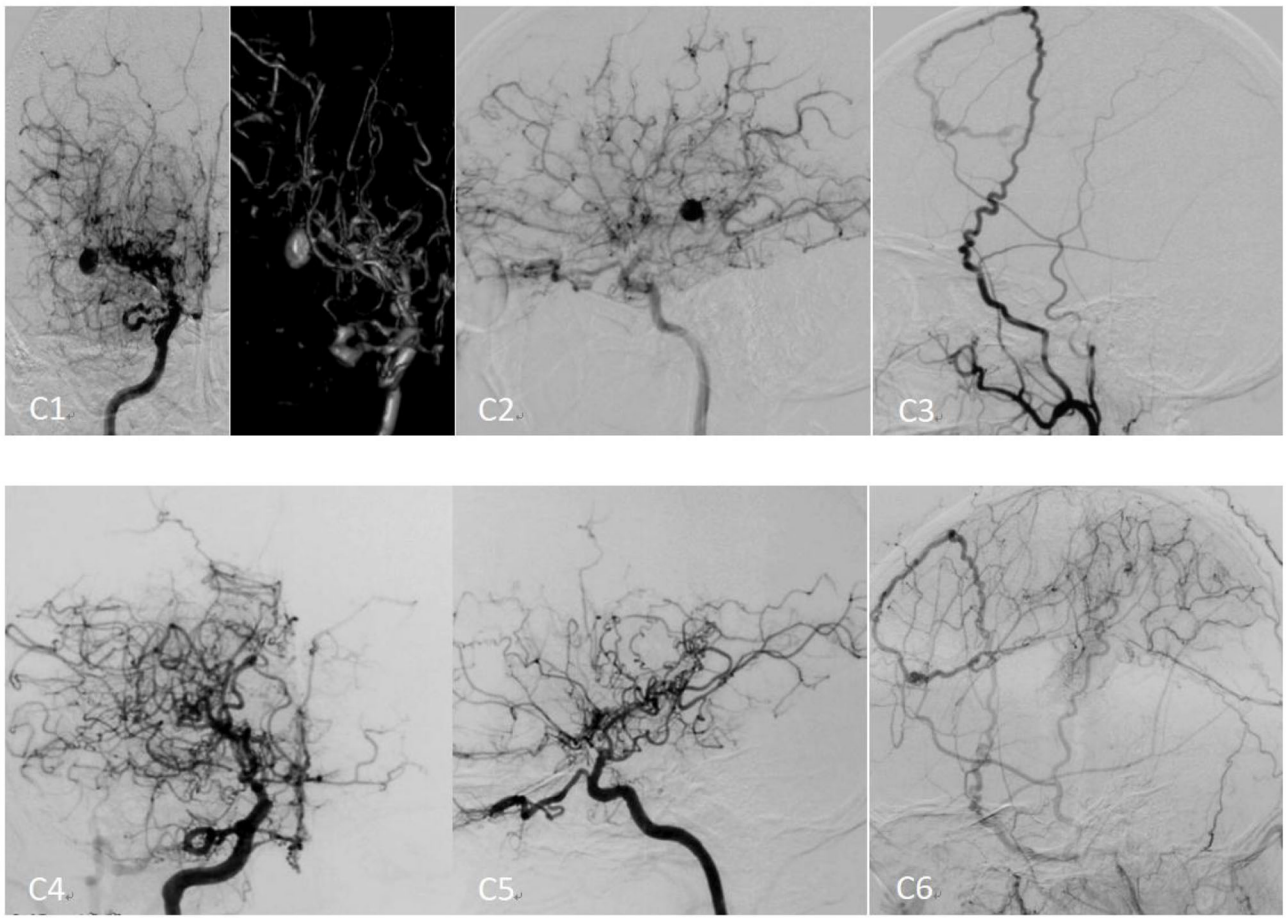
Preoperative and postoperative cerebral angiography of moyamoya disease[[Inline Image]] treated with encephaloduroarteriosynangiosis (EDAS). **(C1)** The preoperative right internal carotid angiography (positive) image. **(C2)** The preoperative right internal carotid angiography (lateral) image. **(C3)** The preoperative right external carotid angiography (lateral) image. **(C4)** The right postoperative internal carotid angiography (positive) image. **(C5)** The postoperative right internal carotid angiography (lateral) image. **(C6)** The postoperative right external carotid angiography (lateral) image.

A total of 19 patients underwent EDAS on the aneurysmal side, among which, nine patients had their aneurysms disappear. In the other eight patients who did not undergo EDAS surgery on the side of the aneurysm, spontaneous aneurysmal disappearance was noted in one patient (Figure 3). Statistical analysis showed that those who underwent EDAS surgery on the aneurysmal side had a higher ratio of aneurysm size or disappearance than those who did not undergo aneurysmal side EDAS surgery. However, this result did not reach statistical significance.

## 4. Discussion

### 4.1. The incidence of MMD combined with intracranial aneurysms

According to epidemiological reports, the overall incidence of intracranial aneurysms is 1%−3%, the ratio of male to female subjects is ~1:1.3, and the average age of onset is 50–69 years (14). Intracranial aneurysms are very rare in children and adolescents younger than 18 years of age (15), accounting for only 0.5%−4.6% of all intracranial aneurysms. It has also been reported that ~3.4%−14.8% of patients with MMD can have intracranial aneurysms (3). The reported incidence of MMD is relatively different, which may be due to the particularly persistent changes in these cases. Some aneurysms in MMD cases will disappear with the progression of the moyamoya disease itself.

### 4.2. Characteristics of MMD combined with intracranial aneurysms

Intracranial aneurysms are abnormal distensions on the walls of the intracranial arteries, which mostly occur in the bifurcation of the intracranial arteries proximal to the Circle of Willis but are quite rare in small peripheral arteries (16). In MMD, pathological changes in the blood vessels are characteristics of intimal fibrocellular hyperplasia, circuitous internal elastic laminae, fractures, and medial atrophy and thinning. With the progression of the disease, damage to the fragile moyamoya vascular elastic layer becomes more pronounced, leading to increased atrophy and a thinner medial wall, microvascularization, and the formation of vascular pathological changes. These pathological changes can be due to local stenosis or occlusion of the vessels of the Circle of Willis (such as the anterior cerebral artery, anterior traffic artery, middle cerebral artery, and internal carotid artery end) causing blood flow shunts. In addition, in MMD, the pathological changes in the Circle of Willis also alter other normal blood vessels (such as the anterior communicating artery, posterior cerebral artery, and basilar artery) and compensatory mechanisms increase blood flow, resulting in increased vessel pressure load, which is more likely to form intracranial aneurysms (17, 18). Therefore, this may also be an important reason why aneurysms commonly occur at the proximal portion of the posterior communicating artery, the posterior cerebral artery, the distal tip of the basilar artery, and the anterior and posterior choroidal artery. As these aneurysms have increased arterial blood flow, they are more likely to rupture and bleed. In this study, there were 35 trunk aneurysms and seven peripheral aneurysms, and the proportion of trunk aneurysms was significantly higher than the peripheral type. Among them, 62.5% of the aneurysms located in the distal part of the anterior circulation and posterior communicating artery decreased in size or disappeared. During the follow-up period, there were two cases of rebleeding events, all in the basal ganglia, and all were not due to ruptured aneurysms, which may be related to the abnormal capillary network of the skull base. Accordingly, the characteristics of moyamoya disease predispose the affected artery to gradually occlude, causing arterial blood flow reduction and leading to a progressive decline in aneurysmal blood flow; thus, the bleeding risk for the corresponding aneurysm is significantly reduced.

### 4.3. Long-term follow-up and treatment of MMD combined with intracranial aneurysms

Intracranial aneurysm rupture causing subarachnoid hemorrhage accounts for ~2%−7% of all strokes, but it accounts for 27% of stroke deaths (16). For the unruptured aneurysm, surgical treatment should be actively performed to avoid rupture and bleeding; however, there is still great controversy about whether the unruptured aneurysm needs surgical treatment (2, 6). The main treatment methods for these are craniotomy clipping and endovascular embolization. However, when the parent artery where the aneurysm is located has undergone vascular stenosis and is completely occluded, and the craniotomy clipping and endovascular embolization treatment cannot be completed, EDAS surgery on the aneurysm side may influence the disappearance of the aneurysm and reduce the chance of aneurysmal rebleeding.

For trunk aneurysms, if the aneurysm is located in the carotid artery distal to another artery, such as the anterior cerebral artery, and if the carrier artery is close to the heart with a vascular lumen featuring uniform stenosis on imaging, the distal parts of the aneurysm may be gradually occluded along with the carrier artery and consequently disappear. Endovascular embolization has been reported to achieve better efficacy in peripheral arterial aneurysms (2, 7). In the treatment of peripheral arterial-type aneurysms that have already ruptured and are actively bleeding, with a relatively thick parent artery, vascular embolization treatment may achieve good results. However, in some patients, wherein the aneurysm-bearing artery is thinner, intravascular embolization may be inadequate as it may participate in the blood supply of a large area or important brain functional areas. Severe neurological dysfunction may occur after the embolization of these kinds of aneurysms, and thus, some aneurysms are not suitable for embolization and should be treated directly (19, 20). For these types of aneurysms, we performed a long-term follow-up study. In this study, the proportion of aneurysms in the anterior circulation was significantly higher than that in the posterior circulation (35:7), and the aneurysms were round or regular in shape, with 81% having a maximum diameter below 5 mm, and 56% in Suzuki stage 3 and 4. During a follow-up period of 20.50 ± 18.28 months, 3.7% of aneurysms were at risk of rupture, 59.3% of aneurysms remained stable, and 37% of them either decreased or disappeared. On follow-up, three out of six peripheral aneurysms were smaller by 50% or had disappeared.

### 4.4. Efficacy of EDAS in MMD combined with intracranial aneurysms

Numerous studies have shown that revascularization can reduce the rate of stroke recurrence and frequency of transient ischemic attacks in patients with MMD, improve their activities of daily living, and activate higher level brain functions (4, 21). Extracranial revascularization of MMD mainly includes direct or indirect extracranial revascularization, or a combination of the two and other techniques. Among these, indirect revascularization is represented by EDAS. In the cases of MMD with untreated aneurysms that we collected, post-EDAS review angiography showed that the aneurysms have disappeared. This may be because after effective revascularization, the pathological hemodynamic trajectory was changed, the abnormal expansion of blood flow pressure was reduced, along with the degree of arterial dilation, and the arterial blood flow velocity and lumen thrombosis were also concurrently reduced, resulting in the disappearance of the aneurysm. It is also possible that the progression of MMD was temporarily accelerated after intracranial blood flow reconstruction, increasing the speed of vascular occlusion and reducing the formation of compensatory vessels, so that the aneurysm disappeared with the parent artery, especially aneurysms in the anterior circulation (22, 23). In this cohort, a total of 19 patients underwent EDAS surgery, including nine patients whose aneurysm disappeared, eight patients without EDAS surgery, and one case without statistical significance, wherein their aneurysms decreased or disappeared after EDAS surgery, confirming that revascularization surgery may have some positive effect on aneurysm reduction or disappearance. In previous studies, EDAS surgery was found to be effective in improving ischemic symptoms (24, 25). Therefore, EDAS surgery can be considered to not only improve cerebral perfusion in patients with MMD but may also promote the disappearance of some aneurysms and reduce the risk of aneurysm rupture and bleeding.

There are several limitations to this study: first, this study was a single-center retrospective study, and selection bias may exist. Second, this study is only based on clinical characteristics, and the natural course of unruptured aneurysms has only been explored from the perspective of imaging; but its pathology and molecular mechanisms have not yet been investigated. Third, the sample size of this study is too small to conduct a subgroup analysis. In further research, we will try to contact more centers to carry out research together, further expand the sample size, and explore the mechanism behind these phenomena.

## Data availability statement

The original contributions presented in the study are included in the article/supplementary material, further inquiries can be directed to the corresponding author.

## Ethics statement

The studies involving human participants were reviewed and approved by the Research Ethics Committee of the Fifth Medical Center of the PLA General Hospital (No. KY-2022-9-69-1). Written informed consent from the patients/participants or patients/participants' legal guardian/next of kin was not required to participate in this study in accordance with the national legislation and the institutional requirements.

## Author contributions

R-MY, CH, and F-BH designed this study. F-BH drafted the manuscript, which was critically revised by all authors and approved by all authors. R-MY and F-BH organized and analyzed the data. All authors conduct the study and the data collection, assisted with interpreting findings, and contributed to the data analysis.
